# Lumbar total disc arthroplasty: outdated surgery or here to stay procedure? A systematic review of current literature

**DOI:** 10.1007/s10195-017-0462-y

**Published:** 2017-07-06

**Authors:** Matteo Formica, Stefano Divano, Luca Cavagnaro, Marco Basso, Andrea Zanirato, Carlo Formica, Lamberto Felli

**Affiliations:** 1 Clinica Ortopedica-IRCCS Azienda Ospedaliera Universitaria San Martino–IST, Istituto Nazionale per la Ricerca sul Cancro, Largo Rosanna Benzi, 10, 16132 GENOVA, GE Italy; 2 IRCCS Istituto Ortopedico Galeazzi, Via Riccardo Galeazzi 4, 20161 MILAN, MI Italy

**Keywords:** Total disc replacement, Lumbar disc arthroplasty, Degenerative disc disease, Outcomes, Complications, Sagittal balance

## Abstract

**Background:**

The purpose of this study was to summarize the available evidence about total lumbar disc replacement (TDR), focusing our attention on four main topics: clinical and functional outcomes, comparison with fusion surgery results, rate of complications and influence on sagittal balance.

**Materials and methods:**

We systematically searched Pubmed, Embase, Medline, Medscape, Google Scholar and Cochrane library databases in order to answer our four main research questions. Effective data were extracted after the assessment of methodological quality of the trials.

**Results:**

Fifty-nine pertinent papers were included. Clinical and functional scores show statistically significant improvements, and they last at all time points compared to baseline. The majority of the articles show there is no significant difference between TDR groups and fusion groups. The literature shows similar rates of complications between the two surgical procedures.

**Conclusions:**

TDR showed significant safety and efficacy, comparable to lumbar fusion. The major advantages of a lumbar TDR over fusion include maintenance of segmental motion and the restoration of the disc height, allowing patients to find their own spinal balance. Disc arthroplasty could be a reliable option in the treatment of degenerative disc disease in years to come.

**Level of evidence:**

II.

## Introduction

Lumbar degenerative disc disease (DDD) is one of the most important causes of low back pain, disability and medical consultations in Western countries and imposes huge economic burdens worldwide.

Most patients suffering from low back pain improve satisfactorily without surgery, but 1–5% of them do not respond to appropriate nonsurgical care, such as muscle strengthening, physical therapy, massage, manipulation, weight control and analgesia, and may be candidates for surgical treatment [1, 2].

Besides surgical techniques, several biological approaches, including the injection of biological substances such as growth factors, bioengineering approaches, and cell or gene therapies have been tested in either preclinical or clinical contexts [3].

Actually, interbody fusion that provides solid anterior support is the gold standard in the treatment of degenerative disc disease.

The fusion of the motion segment eliminates abnormal motion and unburdens loading on pathologic disc tissues, thereby reducing pain and improving quality of life [4].

However, long-term results are sometimes suboptimal in terms of pain relief, and various fusion-related complications such as incorrect placement of screws, breakage of metallic implants, and nonunion have been observed during follow-up for a long time.

Furthermore, there are common surgery complications, such as pseudoarthrosis, with an incidence of 16%, and iliac crest bone graft donor site pain, with an incidence of 9% [5].

Also, adjacent segment disease (ASD) and dissociation between fusion rate and clinical success rate have received more serious attention from surgeons over time [6].

A viable alternative is total disc replacement (TDR), which has increased in popularity in recent decades and has been developed to preserve motion, and possibly reduce adjacent-level degeneration [6].

The aim of our study was to systematically review the available literature on lumbar total disc replacement in patients with chronic low back pain due to DDD, focusing our attention on effectiveness, safety, complication rates and influence of TDR in spinal balance.

## Materials and methods

We performed a systematic review of the available English literature in order to answer four main research questions:

1. What is the evolution of DDD following total disc replacement surgery in terms of pain relief and functional outcomes?
2. What is the effectiveness of total disc replacement surgery compared to other treatments?
3. What is the safety and rate of complications of total disc replacement surgery?
4. How does total disc replacement surgery influence sagittal balance?

The Pubmed, Embase, Medline, Medscape, Google Scholar and Cochrane library databases were screened for relevant studies. The search strategy consisted of a combination of the following keywords: total disc replacement, lumbar disc arthroplasty, degenerative disc disease, outcomes, complications, sagittal balance. We included clinical studies with a follow-up greater than 24 months and with a cohort of patients greater than 20. Only papers related to lumbar total disc replacement were included in our analysis. Non-pertinent manuscripts were excluded. Exclusion criteria were: in vitro studies, case report and review or meta-analysis. We carefully examined reference lists from previous reviews or meta-analysis in order not to miss pertinent papers. The search was limited to studies published in English.

Two reviewers (SD and AZ) independently screened the titles and abstracts from all identified articles to assess their appropriateness to the research focus. In case of conflict among reviewers, a collegial evaluation with remaining authors was performed. References from the identified articles were checked in order not to miss any relevant articles.

**Fig. 1:**
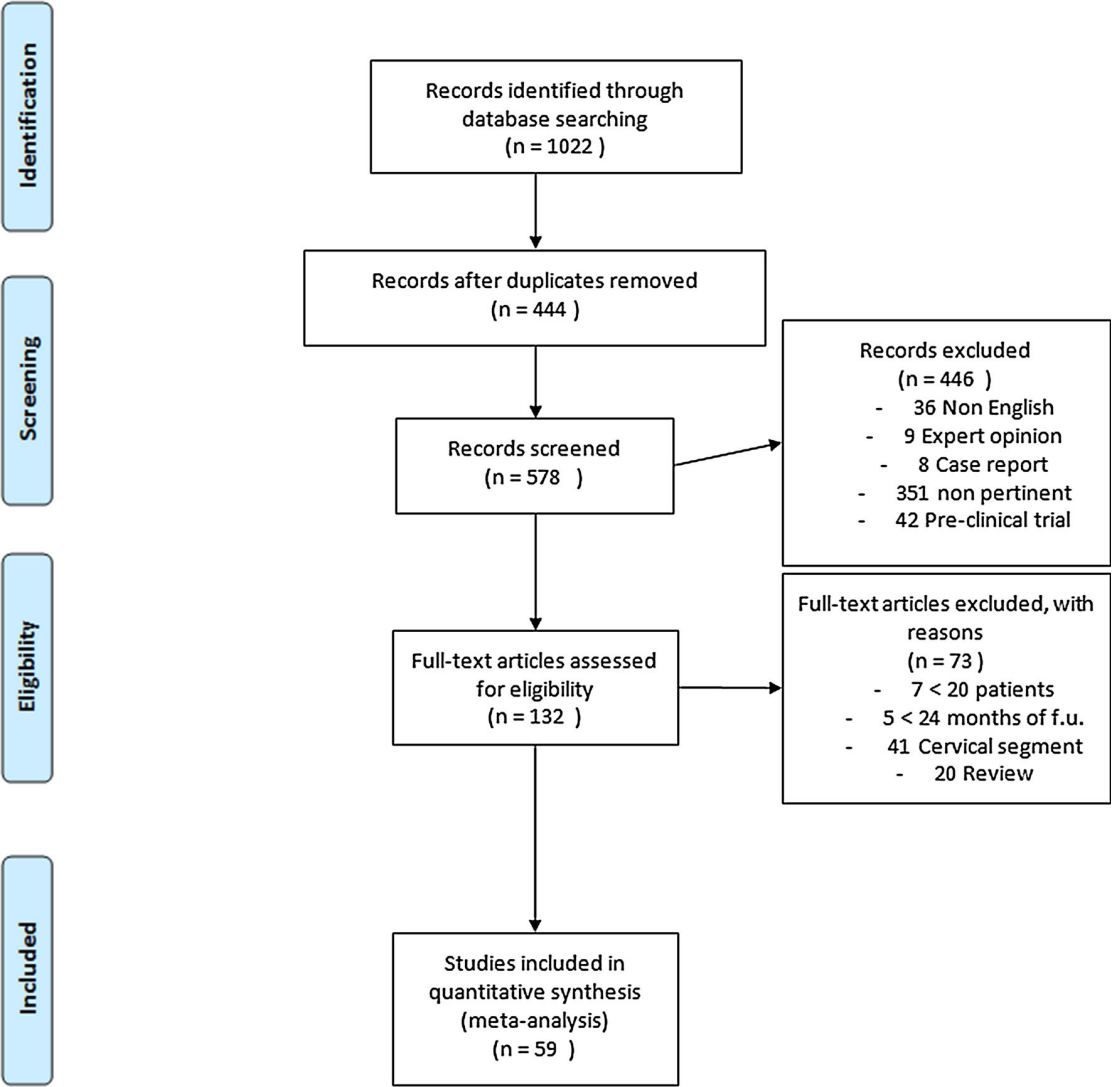
The PRISMA 2009 flow diagram illustrates the review process, the number of the studies identified, included and excluded

All titles and abstracts that met our keywords were examined. The flow diagram illustrates the review process (Fig. 1).

## Results

A total of 1022 articles were identified, 444 duplicates were removed.

Among 578 eligible articles, we selected only those matching our inclusion criteria.

During the selection of papers, no cases of conflict between two authors were reported.

Fifty-nine manuscripts were finally included and fully evaluated. Table 1 summarizes clinical and radiographic outcomes after lumbar TDR in DDD.

**Table 1:** Summary of clinical and radiographic outcomes after lumbar TDR in DDD

References	Year	Study design	EBM level	No. of patients	Average follow-up duration in months	No. of prostheses	Type of prosthesis	Clinical outcomes	Radiographic outcomes	Complications	Implications on sagittal balance	Comparison with fusion surgery
Park et al. [7]	2016	Retrospective case series	2	54	120	69	ProDisc II	VAS and ODI improved significantly	ROM and LL improved only in monosegmental TDR	5 reoperations	–	–
Guyer et al. [8]	2016	Prospective, randomized, controlled, multicenter study	1	394	60	394	Kineflex-L	VAS and ODI improved significantly, 96,8% satisfaction	4° ROM, 0% subsidence, 77.8% HO	24 reoperations	–	–
Garcia et al. [9]	2015	Prospective, multicenter, randomized, controlled study	1	324	24	324	activL	67% ODI and 74% back pain improvement	ROM and disc height improvement, 1.6% HO	6.9% back/leg pain and 1.4% implant subsidence	–	–
Schätz ert al. [10]	2015	Multicenter, single arm, prospective, cohort study	2	83	24	121	M6-L	VAS and ODI improved significantly, no difference between SL and ML	No difference in terms of ROM between SL and ML	–	–	–
Assaker et al. [11]	2015	Prospective, multicenter, observational study	2	134	24	146	Maverick	VAS, ODI and SF-36 improved significantly	>3° of motion (extension–flexion) at the implant level	57 (42%) patients experienced complications	–	–
Lee et al. [12]	2015	Retrospective case series	4	74	60	54	ProDisc-L	–	–	Higher incidence of peritoneal injuries, retrograde ejaculation, superficial abdominal infection	–	Better perioperative outcomes but same revision rate as TLIF
Lu et al. [13]	2015	Retrospective case series	4	35	144	35	Charité III	VAS and ODI improved significantly	ROM significant decrease, IDH no difference, LL significant improvement	71.4% HO, 9.4% subsidence	–	–
Tohmeh et al. [14]	2015	Prospective, multicenter cohort study	2	64	36	64	XL TDR	VAS, ODI and SF-36 improved significantly	IDH increase, 1.6% subsidence, ROM 5.9°, 10.3% HO	No intraoperative complications, no revision surgery	–	–
Lu et al. [15]	2015	Retrospective case series	4	30	29	36	activ-L	VAS and ODI improved significantly	ROM and IDH improved significantly	2 tears of iliac vein, 10% subsidence, 3.3% HO	–	–
Trincat et al. [16]	2015	Retrospective case series	4	108	48	216	ProDisc-L	VAS and ODI improved significantly	ROM improved significantly but less at L5/S1	Complication rate 18%	–	–
Aghayev et al. [17]	2014	Retrospecti- ve case series	4	218	60	305	–	VAS and EQ-5D improved significantly	Average ROM 9.7°, 16.7% grade III HO	Overall 23.4%, intraoperative 4.4%, postoperative 3.2%, revision rate 4%, 10.7% ASD	–	–
Guyer et al. [18]	2014	Prospective, randomized, controlled multicenter study	1	457	24	457	Kineflex-L Disc and Charité	VAS and ODI improved significantly, no difference between 2 groups	ROM improved significantly, ROM >4° in 65.4% vs 62.5%, subsidence 0% vs 0.6%	Revision rate 10.3% vs 8.4%, 71.1% AE	–	–
Siepe et al. [19]	2014	Prospective, single-center clinical investigation of TDR	2	181	89	212	ProDisc II	VAS and ODI improved significantly	–	Complication rate 14.4%, revision rate 7.2%	–	–
Lazennec et al. [20]	2014	Prospective cohort of patients	2	46	24	46	LP-ESP	VAS, ODI and GHQ28 improved significantly	ROM improved significantly, MCR 73% ideal positioning	–	Sagittal balance (SS, PT, SL) did not change significantly at any point of the F-U	–
Strube et al. [21]	2013	Prospective cohort study	2	40	60	40	Maverick	VAS and ODI improved significantly	>Clinical scores correlated with >IDH and >LL	–	–	–
Skold et al. [22]	2013	Prospective randomized controlled trial	1	152	60	115	Charité, ProDisc, Maverick	VAS, ODI, EQ5D and SF36 improved significantly	–	No difference in complication and revision rate between the 2 groups	–	VAS and ODI improved significantly, but less than TDR group
Oktenoglu et al. [23]	2013	Prospective clinical study	2	50	29	25	Maverick	VAS and ODI improved significantly	No difference in terms of LL and segmental lordosis angles	–	–	No difference in radiological outcomes between TDR and TLIF
Meir et al. [24]	2013	Prospective non-randomi zed clinical trial	2	28	116	32	AcroFlex	VAS, ODI, LBOS, SF-36 improved significantly	HO 85%, subsidence 14%	Revision rate 39.3%, ASD 68%	–	–
Zigler et al. [25]	2012	Prospective, randomized, multicenter study	1	236	60	161	ProDisc-L	SF-36, ODI and neurological success improved significantly	ROM preserved and good radiographic outcomes	Revision rate 6.8%, 5.1% AE	–	TDR was not inferior to fusion in terms of effectiveness and safety
Jones et al. [26]	2012	Retrospective case series	4	25	34	31	Charité	OPS and SF36v2 improved significantly	Average DHR 78.3%	–	–	–
Siepe et al. [27]	2012	Prospecrtive cohort study	2	51	50	51	ProDisc II	VAS and ODI improved significantly	Preoperative DSH 6.8mm	–	DDD had a negative correlation with DHS and Pfirmann classification	–
Van de Kelft et al. [28]	2012	Prospective cohort study	2	50	48	50	Maverick	ODI and SF36 improved significantly	Motion was preserved at the operated level	0% revision rate, no major complications	–	–
Park et al. [29]	2012	Retrospective clinical data analysis	4	42	72	51	ProDisc-L	VAS , ODI and SF36 improved significantly	–	–	–	–
Berg et al. [30]	2011	Randomized controlled trial	1	152	24	115	Charité, ProDisc, Maverick	Excellent pain relief in 70% of patients	Motion was preserved in 85% of patients	–	DH and ASD unchanged	Surgical goal was more frequently reached in the TDR group
Scott-Young et al. [31]	2011	Prospective single-center case cohort study	2	122	44.9 ± 23.3	122	Charité	VAS, ODI, SF36 and RMDQ improved significantly	HO 4.9%, optimal placement 94%, average ROM 8.6° ± 3.5°	3.3% revision rate, 0% ASD, subsidence 6.5%	–	–
Blondel et al. [1]	2011	Prospective cohort study	2	221	30	221	–	VAS and ODI improved significantly	Lower scores in patients with Modic 1	9.5% revision rate	–	–
Pettine et al. [32]	2011	Prospective, randomized non- inferiority trial	1	64	24	64	Kineflex Disc and Charité	With both devices VAS and ODI improved significantly	–	0% revision rate	–	–
Rischke et al. [33]	2011	Prospective cohort study	2	50	24	50	Viscoelastic total disc replacement Axiomed	VAS and ODI improved significantly	DH, DA, LL and ROM are maintained	0% device expulsion or fracture	–	–
Pellet [34]	2011	Prospective cohort study	2	99	24	–	Maverick	–	–	–	SSA increased significantly; spine tilt angle was 90°	Significantly more balanced spinal position than ALIF
Katsimihas et al. [35]	2010	Prospective study	2	64	55	64	Charité III	VAS , ODI and SF36 improved significantly	Sagittal rotation 6.5°, subsidence 1.7mm, IT 1.1mm	4.7% early complications, 3.1% revision rate	–	–
Yue et al. [36]	2010	Prospective, randomized, single- masked,	1	414	24	414	Activ-L Disc, Charité and ProDisc-L	VAS and ODI improvement equivalent to control group	ROM conservation equivalent to control group	Safety equivalent to control group	–	–
Siepe et al. [37]	2009	Prospective clinical study	2	161	48	189	ProDisc II	VAS and ODI improved significantly	–	–	–	–
Berg et al. [38]	2009	Prospective, randomised controlled study	1	152	24	80	–	VAS, ODI, SF36 and EQ5D improved significantly	–	Revision rate 10% (mean cause ASD)	–	Effectiveness and safety comparable to fusion group
Sinigaglia et al. [39]	2009	Prospective non- randomized	2	36	39	36	ProDisc II and Maverick	VAS, SF36 and ODI improved significantly	–	Complication rate 80.6%, L4-L5 > L5- S1	–	–
Di Silvestre et al. [40]	2009	Retrospective case series	4	32	36	48	Charité III	VAS, SF36 and ODI improved significantly, with no significant difference between two groups	No significant difference in disc height and ROM improvement between two groups	Complication rate 2-level TDR > 1- level, revision rate 12.5%, no ASD	–	–
Guyer et al. [41]	2009	Randomized controlled trial	1	133	60	90	Charité	VAS, SF36 and ODI improved significantly	ROM, DH, STR improved significantly	–	–	No difference in clinical and radiographic outcomes, TDR has greater rate of employment and lower of long-term disability than ALIF
Guyer et al. [42]	2008	Retrospective case series	4	203	24	203	Charité and ProDisc	Length of time off work is related to VAS and ODI improvement	–	–	–	–
Zigler et al. [43]	2008	Retrospective case series	4	86	24	118	ProDisc	VAS and ODI improved significantly with no difference in two groups	–	-	–	–
Hannibal et al. [44]	2007	Retrospective case series	4	59	24	91	ProDisc	VAS, SF36 and ODI improved significantly with no difference in two groups	–	–	–	–
Zigler et al. [45]	2007	Prospective, randomized, multicenter	1	286	24	211	ProDisc-L	VAS, SF36 and ODI improved significantly	93.7% ROM maintained (average 7.7°)	No major complications	–	Clinical outcomes TDR> fusion
Siepe et al. [46]	2007	Prospective cohort study	2	99	26	119	ProDisc II	VAS and ODI improved significantly, better improvement at L4–L5	–	Complication rate significantly higher in bisegmental TDR	–	–
David et al. [47]	2007	Retrospective clinical and radiographic study	4	106	134	106	Charité	Good result 82.1%, return to work 89.6%	ROM maintained 90.6%, 10.1° and 4.4°	2.8% subsidence, 2.8% ASD with reoperation	–	–
Zigler et al. [48]	2007	prospective, randomized trial	1	157	36	178	ProDisc-L	VAS and ODI improved significantly	–	–	–	No significant difference in clinical outcome between the two groups
Holt et al. [49]	2007	Prospective, randomized, multicenter	1	304	24	205	Charité	–	–	75.6% incidence, 3.4% subsidence, 5.4% revision rate	–	No worse complication rate of TDR than ALIF
Geisler et al. [50]	2007	Multicenter, prospective, randomized	1	304	24	205	Charité	VAS and ODI improved significantly	–	–	–	Better clinical improvement of TDR than ALIF
Tournier et al. [51]	2007	Retrospective case series	3	184	31.2	125	Charité, ProDisc and Maverick	–	ROM improvement <2°, MCR did not depend on the prosthesis Offcentering DH improved but decreased when the prosthesis was offcentered, no difference among type of prostheses	–	PI, PT, SS and TK didn’t change significantly after surgery, LL changed significantly after surgery	–
Siepe et al. [52]	2006	Prospective cohort study	2	92	34.2	108	ProDisc II	VAS, ODI and SF36 improved significantly (better in 1- level TDR)	–	Higher complication rate in bisegmental TDR, overall 19.6%, revision rate 10.9%	–	–
Chung et al. [53]	2006	Prospective cohort study	2	36	24	47	ProDiscII	VAS and ODI improved significantly	DH and ROM improved significantly. Higher postoperative ROM is correlated with better clinical outcome	No major complications	–	–
Chung et al. [54]	2006	Retrospective case series	4	26	30	37	ProDisc	–	The mean ROM at L5-S1 and L4-5 increased significantly from 7.1° to 11.2 ° and from 11.4° to 14.6°	–	LL improved significantly, ST and PT didn’t change significantly	–
Huang et al. [55]	2006	Retrospective radiographic and chart review	4	42	102	60	ProDisc	VAS and ODI are not significantly better in patients without ASD	–	24% ASD	A clear relationship between TDR ROM and the presence of ASD (<5°)	–
Bertagnoli et al. [56]	2006	Prospective non-randomized clinical series	2	104	24	104	ProDisc	VAS and ODI improved significantly in both groups without difference	DH and ROM increased significantly in both groups without difference	–	–	–
Putzier et al. [57]	2006	Retrospective clinical–radiological study	4	71	204	84	Charité	VAS and ODI improved significantly	ASD 17%	Revision rate 11%	–	–
Bertagnoli et al. [58]	2005	Prospective cohort study	2	118	24	118	ProDisc	VAS and ODI improved significantly	DH and ROM increased significantly	No device-related and three approach-related complications	–	–
Bertagnoli et al. [59]	2005	Prospective cohort study	2	25	24	63	ProDisc	VAS and ODI improved significantly	DH and ROM increased significantly	1 case of subsidence, 1 case of anterior extrusion of a polyethylene component	–	–
McAfee et al. [60]	2005	Prospective, randomized, multicenter	1	304	24	205	Charité	Clinical outcomes correlated with surgical technical accuracy	ROM correlated with surgical technical accuracy	Significantly less subsidence in TDR than ALIF	–	ROM and DH improved significantly better in TDR than ALIF
Blumenthal et al. [61]	2005	Prospective, randomized, multicenter	1	304	24	205	Charité	VAS, ODI and SF36 improved significantly	–	Better revision rate for TDR than ALIF (5.4 vs 9.1%)	–	Clinical outcomes, patient satisfaction and hospital stay were significantly better in TDR than ALIF
Lemaire et al. [62]	2005	Retrospective case series	4	100	135	147	Charité	91.6% patients returned to work	2 cases of subsidence, 51.5% DH increased, one case of height loss, mean ROM 10.3° and 5.4°	5 cases of reoperation, 2 neurologic complications, one sexual disfunction, 2 ASD	–	–
Tropiano et al. [63]	2005	Prospective cohort study	2	55	104	78	ProDisc	VAS, ODI and Stauffer- Coventry score improved significantly	No cases of subsidence or DH loss	Seven patients underwent additional surgical procedures, complication rate 9%	–	–
Guyer et al. [64]	2004	Prospective randomized clinical trial	1	144	24	100	Charité	VAS and ODI improved significantly in both groups	No subsidence, 1 case of HO	Three patients underwent additional surgical procedures	–	No significant difference in effectiveness and safety between TDR and BAK cages

*TK* thoracic kyphosis, *ST* sacral tilt, *ST* segmental translation, *DA* disc angle, *SSA* spino-sacral angle, *IT* intervertebral translation, *MCR* mean center of rotation, *LBOS* low back outcome scores, *VAS* visual analogue scale, *ODI* oswestry disability index, *ROM* range of motion, *BAK* bagby and kuslich implant

### What is the evolution of DDD following total disc replacement surgery in terms of pain relief and functional outcomes?

Total VAS and ODI scores statistically decreased from preoperative to 1–2 years after surgery.

Although these scores increased until the last follow-up, they remained significantly lower than the preoperative values.

Schätz et al. [10] reported no significant differences, in terms of VAS and ODI improvement, between single-level and multi-level subgroups.

On the other hand, Siepe et al. [46] observed that postoperative outcome was significantly inferior following bisegmental disc replacements at L4–L5/L5–S1 with a considerably higher complication rate when compared with monosegmental TDR procedures.

Moreover, they highlighted VAS and ODI deterioration when disc replacement was performed at the lumbosacral junction, while most of other articles do not show difference depending on operated level.

Tohmeh et al. [14] showed there was a significant reduction in medication usage from baseline to last follow-up.

Ziegler et al. [25] examined neurological status, defined as the maintenance or improvement of patient responses to all neurological criteria: sensory and motor status, reflexes, and straight-leg test.

At 2 years of follow-up, the TDR group was statistically superior to the fusion group, with 91.2% success (135 of 148 patients) compared to 81.4% (57 of 70 patients), respectively.

The literature suggests that there is no significant difference, in terms of clinical outcomes, between various prostheses.

Pettine et al. [32] showed similar improvement, in terms of VAS and ODI scores for the Kineflex Disc group and Charité group at 2 years of follow-up (56.80, 37.30 and 54.43, 38.40, respectively).

David et al. [47] showed how 89.6% of patients returned to work after surgery, including 77.8% of patients working in hard labor employment, and 96.7% working in sedentary or light duty employment before surgery.

The correct positioning of TDR is crucial. McAfee et al. [60] showed us that mean ODI and VAS scores improved with the degree of technical accuracy.

In conclusion, many studies suggest pain relief, improvement in functional status and patient satisfaction after TDR surgery.

Unfortunately, detailed information about outcome measurement is often lacking. Moreover, the majority of the included studies were uncontrolled ones. Indeed, the quality of these studies is not sufficient to draw definite conclusions about pain relief and functional outcomes after TDR surgery.

### What is the effectiveness of total disc replacement surgery compared to other treatments?

Although TDR achieved optimal outcomes, it is essential to compare these results with the outcomes obtained with the gold standard technique (fusion surgery).

Nearly every work shows similar patterns of two main clinical parameters, VAS and ODI scores: both techniques offered significant improvements.

This improvement lasts at all time points compared to baseline. The majority of the articles show there is no difference in terms of clinical outcome between the two groups.

Ziegler et al. [25] demonstrated that both TDR and fusion treatment groups obtain significant improvement in ODI at 5 years compared with baseline. VAS pain scores decreased from preoperative values by 48% in both treatment groups at 5 years. Patients were highly satisfied in both groups (77%).

On the other hand, some articles underline a better clinical trend in the TDR group, although both surgical techniques lead to satisfying results. Skold et al. [22] conclude that significant differences in favour of TDR concerning back pain, pain improvement, and ODI were present at 1 year and disappeared at 2 years, but reappeared at the 5-year follow-up.

Trying to analyze functional outcomes, Guyer et al. [41] summarized that full-time employment was achieved by 65.6% of patients in the Charité, group versus 46.5% of patients in the BAK group.

Similarly, the rate of long-term disability (8 vs 20.9%) achieved a statistically significant difference between groups. No significant difference was observed for all other parameters.

Oktenoglu et al. [23] showed that there was a statistically significant difference between the levels of blood loss in the two groups: the level of blood loss was significantly higher in the TDR group compared to the PTDS (posterior transpedicular dynamic stabilization) group. Furthermore, operation time and length of hospital stay were significantly longer in the TDR group compared to the posterior dynamic stabilization group.

Blumenthal et al. [61], by contrast, reported the hospital stay was significantly shorter in the Charité™ artificial disc group.

Another interesting aspect is the radiographical outcome in terms of spontaneous fusion, range of motion at the operated and adjacent level, postoperative disc height, rate of subsidence and spinopelvic parameters.

Ziegler et al. [25] reported that none of the TDRs developed spontaneous fusion. The segmental range of motion following TDR remained within normal range.

Oktenoglu et al. [23] showed comparable results in postoperative radiographic evaluation for both techniques (TDR and PTDS). Both dynamic systems provided spine stability.

McAfee et al. [60] noted that TDR patients had a 13.6% mean increase in mean flexion/extension ROM at 24 months postoperatively compared to baseline. The control group showed an 82.5% decrease in the same parameter. Besides, patients in the TDR group had significantly better restoration of disc height and less subsidence.

Berg et al. [30] found different results. The preoperative flexion–extension ROM was similar between the fusion and TDR groups, and preoperative disc heights of segments to be treated were between one and two standard deviations less than that previously established in a normative database. Seventy percent of fused patients had no mobility, whereas 85% of TDR patients were mobile at 24 months of follow-up. Moreover, they noticed significant differences at adjacent segments, with more translation and flexion–extension in the fusion group rather than in the TDR group.

Auerbach et al. [65] also analyzed the differences between TDR and fusion in terms of ROM. They found no preoperative differences at the L4/L5 or L5/S1 operative levels. At 24 months after surgery, within-group comparisons revealed a statistically significant increase in total lumbar ROM only in the group undergoing TDR at L4/5, while there were no significant differences within the groups undergoing fusions at L4/5, fusions at L5/S1, or TDR at L5/S1. Between-group comparisons revealed no significant differences. Segmental contribution to total lumbar ROM was significantly reduced at the operative level for fusions at both L4/5 and L5/S1. In the TDR group, segmental ROM at the operative level was reduced at L5/S1 and relatively preserved when the operative level was L4/5. Segmental contribution to total lumbar ROM was significantly reduced at the operative level for fusions at both L4/5 and L5/S1. In the TDR group, segmental ROM at the operative level was reduced at L5/S1 and relatively preserved when the operative level was L4/5.

Fusion at L5/S1 was associated with a significant increase in segmental contribution to ROM at the first cranial adjacent level, with insignificant increases at each subsequent cranial adjacent level. The same was true for fusion at L4/L5 but this increase was not statistically significant. TDR at L4/5 was associated with small but significant increases in segmental ROM at the first cranial and caudal adjacent levels. TDR at L5/S1 did not result in a change in ROM at the first cranial adjacent level, but was associated with a significant increase in ROM at the second cranial adjacent level. TDR or fusion at whatever operative level did not result in significant changes in segmental ROM at cranial or caudal non-adjacent levels over the follow-up period.

Again, Oktenoglu et al. [23] reported that there were no significant differences observed between the preoperative and postoperative lumbar (LL) and segmental lordosis (SL) evaluations for both techniques.

Finally, Pellet et al. [34] evaluated TDR in terms of spinal balance. They observed that SSA (spinosacral angle) was considerably increased in the discal arthroplasty group, resulting in a significantly more balanced spinal position.

In the group of patients undergoing arthrodesis using the ALIF technique, no such significant improvement was found, despite the use of a lordotic cage.

They showed that in cases of low pelvic incidence, it was necessary to maintain a Roussouly type 1 or 2 back without increasing lordosis. Indeed, L4–L5 disc prostheses is a valuable approach in these subjects. L5–S1 arthrodesis seemed a more suitable approach for treating patients with elevated sacral slope (back type 3 or 4).

### What is the safety and rate of complications of total disc replacement surgery?

The literature shows similar rates of complications between TDR and fusion procedures.

Lee et al. [12] noted that there was a trend toward more surgical-approach-related complications in the TDR group (16.7%) compared to the TLIF group (5.0%). The higher surgical-approach complication rate could be due to the steep learning curve of TDR surgery.

Holt et al. [49] observed no differences in terms of complication rate, also reporting a reoperation rate of 5.4% in the TDR group and of 9.1% in the fusion group, which is a significant difference.

The same result was found by Guyer et al. [41], with additional index-level surgery performed in 7.7% of Charité patients and 16.3% of BAK patients.

Unfortunately is difficult to compare results because there are lots of confounding factors (e.g. type of prosthesis, sample size, epidemiological features, surgical experience). However, the main certainty seems to be that there are no significant differences, in terms of rate of complications and reoperation, between TDR and fusion techniques. In Table 2 we summarize rates and types of complications occurring in the examined papers.

**Table 2:** Summary of rates and types of complications occurring in the examined papers

References	Reoperation	Mean cause of reoperation	Other complications	HO	Subsi-dence	Adjacent segment disease	Overall
Park et al. [7]	5 (9.3%)	Degenerative spondylolisthesis and facet arthritis	–	–	–	–	–
Guyer et al. [8]	24 (11.8%)	Stenosis	–	15.9%	0%	–	–
Garcia et al. [9]	2.3%	Pain	–	1.6%	1%	–	30%
Assaker et al. [11]	4 (3%)	Abdominal wall weakness	–	–	–	–	42% (57)
Lee et al. [12]	4 (10.5%)	Facet arthritis	Peritoneal injuries, abdominal infection and retrograde ejaculation	–	–	–	27.2%
Lu et al. [13]	–	–	Leg pain, pedicle fracture, tear of iliac vein, anhidrosis and abdominal hernia	71.4%	3 (9.4%)	–	–
Tohmeh et al. [14]	0 (0%)	–	–	3 (5.4%)	1 (1.6) %	–	–
Lu et al. [15]	0 (0%)	–	Tear of iliac vein	1 (3.3%)	3 (10%)	–	–
Aghayev et al. [17]	10 (4%)	Implant dislocation	Vessel injuries, dura lesions, vertebral fracture, ureter lesions	–	–	11 (10.7%)	23.4%
Guyer et al. [18]	10.3%	Stenosis	–	–	0%	–	71.1%
Siepe et al. [19]	34 (17%)	Adjacent level disc herniation	Postsympathectomy syndrome, retrograde ejaculation, abdominal hematoma	–	–	–	–
Skold et al. [22]	5 (6.3%)	Hernia	Suspected facet joint pain, hematoma, nerve entrapment, meralgia parestheti-ca	–	1	–	–
Meir et al. [24]	11(39.3%)	Device failure	Pain, tear of iliac vein	12 (85.7%)	–	68%	–
Scott-Young et al. [31]	4 (3.3%)	Device dislocation	Wound infection, nerve irritation, spondylolisthesis, discogenic pain	–	6.5%	0%	–
Blondel et al. [1]	21 (9.5%)	Persistent pain	Vascular lesions, retrograde ejaculation, impaction of a keel, wound hematoma	–	–	5 (2.25%)	–
Katsimihas et al. [35]	3 (4.7%)	–	Retroperitoneal hematoma, superficial abdominal hematoma, retrograde ejaculation	–	44 (83%)	1 (1.5%)	–
Berg et al. [38]	8 (10%)	Recurrent pain	Hematoma, nerve entrapment, wound hernia, meralgia paresthetica, dural tear	–	1	1	17.5%
Sinigaglia et al. [39]	–	–	Laparoceles, persistent abdominal pain, wound dehiscence, urinary disorder, paresthesia, radiculitis	0%	–	–	80.6%
Di Silvestre et al. [40]	2 (12.5%)	–	Tear of iliac vein, severe anemia, persistent sciatica	–	1 (6.25%)	–	–
Siepe et al. [46]	8 (8.1%)	–	Abdominal wall hematoma, dislocation	1	–	1	17 (17.2%)
David et al. [47]	11 (10.4%)	Symptomatic facet arthrosis	Nerve irritation, core dislocation, adjacent disc herniation	–	3 (2.8%)	3 (2.8%)	–
Holt et al. [49]	11 (5.4%)	–	Venous injury, retrograde ejaculation, ileus, vein thrombosis, blood loss, incisional hernia, epidural hematoma, dural tear, infection, neurological complications, stenosis, spondylolisthesis	–	7	2	155 (75.6%)
Siepe et al. [52]	10.9%	–	Retrograde ejaculation, sympathectomy related dysesthesia, extraforaminal disc protrusion,	1	2	2	18 (19.6%)
Putzier et al. [57]	5 (9%)	Implant subsidence	Implant fracture, implant dislocation, persistent pain	–	2	9 (17%)	–
Bertagnoli et al. [58]	–	–	Peritoneal hematoma, superficial hematoma, retrograde ejaculation, persistent leg pain	–	0	–	–
Lemaire et al. [62]	5 (5%)		Symptomatic articular arthritis, retrograde ejaculation, acute leg ischemia, vascular injuries, neurological complications	3	2	2	–
Tropiano et al. [63]	7	–	Deep venous thrombosis, iliac vein laceration, retrograde ejaculation, incisional hernia, radicular pain	–	0	–	9%

### How does total disc replacement surgery influence sagittal balance?

The implantation of a total disc arthroplasty can induce changes in spinal balance. Lazennec et al. [20] reported that only the SL significantly increased for about 10° after implantation and remained stable afterward while variations in SS (sacral slope) and PT (pelvic tilt) were not significant. At the instrumented level, the mean center of rotation (MCR) location was physiological in 70% of mobile cases before surgery, 76% at 12 months, and 73% at 24 months and at the upper adjacent level in 89, 100, and 90% of cases, respectively. The average ROM in flexion/extension at 2-year follow-up was 5.4° and 64.2°; 66% of cases were mobile at 12 months and 76% at 24 months. The ROM of the replaced disc and the adjacent upper level did not change significantly between different time points.

Huang et al. [55] underline a clear relationship between TDR ROM and the presence of ASD at 8.6-year follow-up: the patients with ASD had a ROM of 1.6° and 61.3° whereas the patients without ASD had ROM 4.7° and 64.5°.

In fact, when patients were stratified by ROM, no patients with ROM 5° or greater developed ASD. When patients were divided according to ROM (5° or greater, and less than 5°), the prevalence of ASD was 0% in the high ROM group and 34% in the low ROM group. Similarly, in patients with ASD, 100% had ROM less than 5°. In patients without ASD, 59% had ROM less than 5°.

In the study by Chung et al. [54] the mean sagittal ROM at each operative segment increased significantly from 7.1° to 11.2° and from 11.4° to 14.6° at the L5–S1 and L4–5 levels, respectively.

In all patients who underwent a single- or double-level TDR, the mean LL and SL at L4–5 level increased significantly, while an analysis of the changes in the ST (sacral tilt), PT and SL at L1–2, L2–3, L3–4, and L5–S1 levels did not show significant differences.

Among patients who underwent a single-level TDR at the L4-5 level, the mean SL at the L4–5 operative level and the mean LL increased significantly and there was no significant difference in the ST, PT, and the SL at the L1–2, L2–3, L3–4, and L5–S1 levels.

In patients who underwent a single-level TDR at the L5-S1 level, the mean SL at the L5–S1 level increased significantly. The LL showed a similar trend to that of the single-level TDR at the L4-5 level, but there was no statistical significance.

No significant difference was detected for the ST, PT, and the SL at the L1–2, L2–3, L3–4, and L4–5 levels.

Le Huec et al. [66] reported that the changes in global lordosis, SS, and PT were not significant in patients undergoing a single-level TDR. Additionally, there was no significant difference in the preoperative and postoperative values of kyphosis, segmental lordosis of L4–L5, or L5–S1. There was no statistical difference with regard to the overall lordosis, SS, PT, or kyphosis when the two groups were compared with each other.

However, if we consider only the L4–L5 group, the segmental lordosis was significantly increased after the total disc arthroplasty. The same results were obtained in the L5–S1 group. While the prosthesis increased lordosis at the level implanted, the overall lordosis did not change, thus indicating the adaptability of the spine as a whole to maintain lordosis. Furthermore, an angular change of more than 3° was observed in all patients with average motion of 6.5° (7.3° and 5.2° at L4-L5 and L5-S1, respectively).

Pellet et al. [34] made an important contribution to this topic. They reported that the spinosacral angle (SSA) increases significantly after disc arthroplasty, resulting in a more balanced spinal position. The C7 plumb line shifted behind the posterior superior corner of S1 and became negative in the majority of patients. The authors observed a significant increase in SSA among patients undergoing L5-S1 arthroplasty, as well as backward displacement of the C7 plumb line. In the L4–L5 group there was a non-statistically significant increase in SSA, while the postoperative plumb line had moved behind the posterior superior angle at S1.

This paper underlines a clear difference in the 4 back types (according to Roussouly’s classification) in terms of spinopelvic parameters (pelvic incidence PI, PT, SS and SSA) but not of balance parameters (S1-C7, hip axis and S1 vertebra and C7 ratio). The difference in terms of SSA found preoperatively between the different back types was not seen postoperatively. The SSA was highly correlated with PI, SS and distal LL; it was negatively correlated with the C7 plumb line.

Finally, Tournier et al. [51] explored every spinopelvic parameter separately. The mean PI is not different before and after disc replacement. The same happens for pelvic tilt: only 89% of the patients were in the normal range. The authors found an improvement only after L5-S1 prosthesis. Nearly 92% of the patients had a normal SS before surgery, 94.2% after TDR. The mean SS improves after L5–S1 prosthesis (from 35.4° to 36.3°) and L4–L5 prosthesis (from 36.2° to 37.4°).

The mean lumbar lordosis in the total sample is significantly higher after total disc replacement. The increase of L1–S1 lordosis is neither linked with an increased angle at the prosthesis level, nor with an increased ROM at the prosthesis level. Almost 94% of the patients have a postoperative LL in the physiological range. The L1–S1 lordosis is associated neither with the sagittal prosthesis centring, nor with prosthesis size. The lumbar curvature depends on the prosthesis level: L4–S1 curvature represents 93% of the total LL after L3–L4 prosthesis, and 73% of the total LL after L4–L5 and L5–S1 prostheses. The mean thoracic kyphosis (T4–T12) is 37° before surgery and 36.7° after total disc arthroplasty. The difference is not significant.

## Discussion

Lumbar fusion, including traditional techniques with different approaches, is a well-established surgical technique for the treatment of degenerative disc diseases [67, 68].

Even if clinical outcomes are satisfactory and lead to well-known benefits, the original biomechanics of the spine is altered because of the lack of motion at the fused segments. In addition, spinal fusion is burdened by a not negligible rate of adjacent segment degeneration. TDR has increased in popularity as an alternative for lumbar fusion. The technique aims to restore and maintain spinal segment motion, attempting to prevent adjacent level degeneration at upper or lower segments.

Certainly, there is still debate on the preferred surgical technique, because TDR cannot be considered a complication-free procedure. Moreover, the increasing attention given to spinal balance allows the evaluation of TDR according to this new perspective. The main focus for establishing the ideal surgical technique is clinical outcome.

Most papers show significant effectiveness of TDR in terms of improvement in all clinical scores. Along with clinical aspects, blood loss, hospital stay, length of surgery and medication use have been evaluated. TDR shows significant superiority in shortened duration of hospitalization when compared to fusion techniques. There was no significant difference in operation time, blood loss, complications, reoperation rate and proportion of patients who returned to full-time/part-time work between the TDR group and the fusion group. Of course, different fusion procedures and different types of artificial discs may represent biases in comparing outcomes. In addition, the results are affected by heterogeneity caused by random sampling and different epidemiological features.

In most of the included articles, there is no clear and general consensus about the indications of these two surgical procedures. However, it is well known that fusion surgery indications are wider than TDR ones. Anyway, especially for young patients suffering from DDD without any significant instability, deformity or osteoporosis, TDR might be a suitable alternative to lumbar fusion.

In terms of safety, TDR shows some differences in comparison to fusion techniques. While there are no significant differences in overall rate of complications or reoperation, there is some diversity when we analyze the types of complications.

It is universally accepted that the main limits of the fusion technique are loss of motion at the operative level, and adjacent segment degeneration.

There is moderate evidence to suggest that patients who undergo fusion may be nearly 6 times more likely to be treated for ASD than those who undergo TDR. From 2 randomized trials, the pooled risk of clinical ASD treated surgically was 1.2 and 7.0% in the TDR and fusion groups, respectively [69].

While TDR restores spinal segment motion, it is burdened by the same kinds of complications that affect an anterior approach to the spine. That’s why we are not surprised to find a higher rate of access-related complications in comparison to fusion surgery, with a not negligible number of great vessel injuries, abdominal wall lesions and retrograde ejaculation. The rate of surgical-approach-related complications in the ADR group was 16.7%, while that in the TLIF group was 5.0%. Complications included peritoneal injuries (*n* = 5; 9.3%), superficial abdominal infection (*n* = 3; 5.6%) and retrograde ejaculation (*n* = 1; 1.9%) [12].

The major advantages of a lumbar TDR over fusion include the maintenance of segmental motion and the restoration of the disc height. These two features became fundamental when we correlate TDR outcomes according a spinal balance evaluation.

Sagittal balance has to be considered in every spinal surgical procedure. Surgical correction of this parameter, especially when heavily impaired, is mandatory and often affected by severe complications due to the complexity of the procedure itself [70].

Most of the analyzed papers show that the variables in the patient population with degenerative disc disease are similar to those of asymptomatic individuals. However, several surgical treatments, including spinal fusions, can deleteriously alter the sagittal balance.

The tendency towards normalization of the alterations of sagittal balance, or at least maintaining it, confirmed the regulatory role of total disc arthroplasty, which allows patients to position themselves appropriately. This motion preserving technique refurbishes the compensatory mechanisms at the operated segment, allowing patients to find their own spinal balance. In order to achieve these goals, the correct positioning of the prosthesis in terms of size and mean center of rotation is of paramount importance.

In conclusion, although further studies with larger groups of patients and a longer follow-up period is needed to better evaluate the outcomes and safety of lumbar TDR, it seems clear that disc arthroplasty could be a reliable option in the treatment of degenerative disc disease in years to come.
